# Immunosuppression in Sepsis: Biomarkers and Specialized Pro-Resolving Mediators

**DOI:** 10.3390/biomedicines12010175

**Published:** 2024-01-13

**Authors:** Cristina M. Padovani, Kingsley Yin

**Affiliations:** Department of Cell Biology and Neuroscience, Rowan-Virtua School of Translational Biomedical Engineering and Sciences, Virtua Health College of Life Sciences of Rowan University, Stratford, NJ 08084, USA; yinki@rowan.edu

**Keywords:** macrophages, lymphocytes, apoptosis, infection, MDSCs, biomarkers, sepsis, SPMs

## Abstract

Severe infection can lead to sepsis. In sepsis, the host mounts an inappropriately large inflammatory response in an attempt to clear the invading pathogen. This sustained high level of inflammation may cause tissue injury and organ failure. Later in sepsis, a paradoxical immunosuppression occurs, where the host is unable to clear the preexisting infection and is susceptible to secondary infections. A major issue with sepsis treatment is that it is difficult for physicians to ascertain which stage of sepsis the patient is in. Sepsis treatment will depend on the patient’s immune status across the spectrum of the disease, and these immune statuses are nearly polar opposites in the early and late stages of sepsis. Furthermore, there is no approved treatment that can resolve inflammation without contributing to immunosuppression within the host. Here, we review the major mechanisms of sepsis-induced immunosuppression and the biomarkers of the immunosuppressive phase of sepsis. We focused on reviewing three main mechanisms of immunosuppression in sepsis. These are lymphocyte apoptosis, monocyte/macrophage exhaustion, and increased migration of myeloid-derived suppressor cells (MDSCs). The biomarkers of septic immunosuppression that we discuss include increased MDSC production/migration and IL-10 levels, decreased lymphocyte counts and HLA-DR expression, and increased GPR18 expression. We also review the literature on the use of specialized pro-resolving mediators (SPMs) in different models of infection and/or sepsis, as these compounds have been reported to resolve inflammation without being immunosuppressive. To obtain the necessary information, we searched the PubMed database using the keywords sepsis, lymphocyte apoptosis, macrophage exhaustion, MDSCs, biomarkers, and SPMs.

## 1. Introduction

During infection, the immune system initiates a complex and tightly coordinated response not only to clear the host of the infection, but also to ensure it will be prepared the next time it encounters the same pathogen. The host’s immune system utilizes a highly regulated balance of pro-inflammatory, anti-inflammatory, and resolution processes in order to achieve this. If one of these processes becomes dysregulated, the delicate homeostatic balance will become disrupted, ultimately leading to chronic disease and/or death.

Sepsis is defined as a life-threatening infection that can lead to organ failure. It is the leading cause of death in U.S. hospitals, and its high mortality rate may be attributed to a lack of diagnostics and viable treatment regimens. Sepsis management typically focuses on blunting the early, hyper-proinflammatory phase of sepsis, where the patient’s tissues and organs are overwhelmed by a release of cytokines—a phenomenon known as cytokine release syndrome (CRS). CRS is a clinical syndrome that comprises the early phase of sepsis. CRS can be defined as a hyper-proinflammatory immune response to a stimulus leading to elevated levels of pro-inflammatory cytokines, inflammation, and organ dysfunction [1]. There is significant overlap between this definition and the consensus definition of the early phase of sepsis; however, this early phase typically is followed by a late phase of immunosuppression [1]. While it is reported that states of severe immunosuppression predispose a patient to developing CRS, this association does not appear to always work in the reverse [1]. There is suggestion that CRS and sepsis could be linked, but this connection has not been completely confirmed [1,2].

Historically, there was very little focus on management of the late, immunosuppressive phase of sepsis. In this stage, the patient’s immune system is considered to be hypoactive, as these deaths are attributed to the patient being unable to clear the original infection or to the acquisition of a secondary infection [3]. Better treatments and strategies for sepsis management are necessary; however, before effective modalities targeting the late phase of sepsis can be implemented, there needs to be a better understanding of the factors contributing to sepsis-induced immunosuppression.

Here, we review current knowledge of immunosuppression in sepsis. We describe three major hallmarks of sepsis-induced immunosuppression (lymphocyte apoptosis, monocyte/macrophage exhaustion, and myeloid-derived suppressor cell migration), with a particular focus on the dysregulation of immune cell responses. In addition to this, we compare two highly debated phenomena that both appear to occur at least in part in septic patients: endotoxin tolerance and monocyte/macrophage exhaustion.

At present, there is no one treatment that has consistently been shown to reduce morbidity and mortality in sepsis patients, despite decades of research and hundreds of clinical trials [4]. Even though there have been recent advancements in antibiotic therapy, there is still a need for new, effective treatments for sepsis. We argue that successful treatment in sepsis patients relies on physician understanding of the patient’s immune status, which is highly dependent on what stage of sepsis the patient is in, and what therapeutics would therefore be the most effective. These therapeutics rely on biomarkers that may enable real-time immunological profiling of individual sepsis patients, especially those in the late, immunosuppressive phase. We will review accepted and potential biomarkers that could aid physicians in delineating their patients’ immune status during late sepsis.

## 2. Methods

### 2.1. Search Term Strategy

From October 2023 to January 2024, research studies were identified by searching the research database PubMed. The following string of search terms was used to identify peer-reviewed articles in this database: “immunosuppression” AND “sepsis”; “sepsis” AND “monocytes OR macrophages”; “sepsis” AND “lymphocyte apoptosis”; “sepsis” AND “myeloid-derived suppressor cells OR MDSCs”; “sepsis” AND “biomarkers”; sepsis AND “specialized pro-resolving mediators OR SPMs OR Resolvin OR Lipoxin”. A reference search was conducted using the final selected articles.

### 2.2. Inclusion Criteria

Types of studies. The studies that are included contained mostly mixed data research, primarily composed of hypothesis-driven benchtop research, prospective cohort studies, and observational studies. Only articles that were written in the English language were evaluated.

Types of participants. Reviewed studies included human participants who met the following criteria: males and females; patients who were diagnosed with sepsis according to that hospital’s standards (typically by using sepsis-3 criteria); living in the U.S., not including U.S. territories; patients without an active diagnosis of sepsis (control group). The racial background and age of participants were not used as selection criteria. Reviewed studies also included preclinical animal models of sepsis and infection.

### 2.3. Exclusion Criteria

While we tried to include mostly articles that were published within the past 10 years, no research articles were excluded on the basis of published year alone.

### 2.4. Data Extraction

Data extracted from the final selected articles included article information, methodology, and results of the studies. Information was collected on the article such as title, authors, and published year. Data on methodology included demographics of the study population (if applicable), study design, sample size, qualitative aspects such as tissue appearance for mouse experiments, and quantitative aspects such as patient laboratory results and statistical significance.

### 2.5. Data Analysis

Extracted data were analyzed quantitatively and descriptively. A review of the results was run by the researchers to provide statistical significance across references.

## 3. The Innate Immune System and Inflammatory Response in Infection

**Figure 1:**
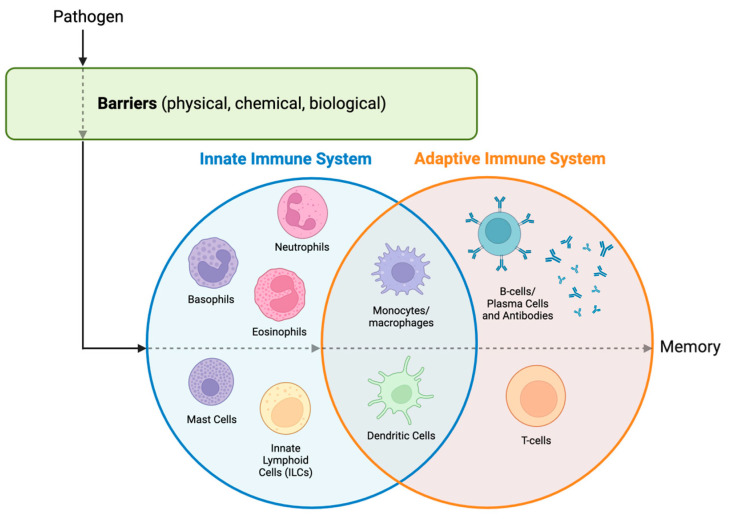
Barriers of defense. Once a pathogen breaches the first line of defense (host barriers), it is then encountered by the innate immune system. Main cell players in the innate immune system include neutrophils, basophils, eosinophils, mast cells, and innate lymphoid cells (ILCs; e.g., natural killer cells) [5,6,7]. If the innate immune system is not successful at completely clearing this pathogen, the pathogen needs to contend with the adaptive immune system. Main cell players in the adaptive immune system include B cells, plasma cells, and T cells [5]. Monocytes/macrophages and dendritic cells are innate immune cells that are said to bridge both the innate and adaptive immune systems, as they are the main initiators of the adaptive immune system [8]. Once the adaptive immune system successfully removes the pathogen, immunological memory is established. Key: defense breach is depicted by a dotted arrow. Created in BioRender.com.

Levels of defense against pathogens include host barriers and the human immune system [5]. Host barriers include physical, chemical, and biological obstacles that help prevent the entrance of pathogens. Physical barriers include the skin and mucous membranes, chemical barriers include pH and lysozymes, and biological barriers include the host’s normal flora [5]. If these barriers are breached, then the pathogen will encounter the host’s immune system. The human immune system can be subdivided into two systems: the innate immune system and the adaptive immune system. The innate immune response is quick-acting, does not discriminate, and does not develop memory, whereas the adaptive immune response is slow-acting, is highly specific, and develops memory (See Table 1 and Figure 1) [6].

The innate immune response acts first and is usually strong enough to clear an infection without triggering the adaptive immune response. A key feature of this immune system is that it must be able to differentiate between “self” and “non-self” so that it does not accidentally attack the body’s own cells, thinking they are pathogens [6]. It is able to perform such discrimination via recognition of pathogen-associated molecular patterns (PAMPs), conserved motifs found on a pathogen’s surfaces, which help the immune system identify the pathogen as foreign, or “non-self” [6]. Some examples of PAMPs are lipopolysaccharide (LPS) found in the outer membranes of gram-negative bacteria, peptidoglycans found in gram-negative and gram-positive bacterial cell walls, lipoteichoic acid from gram-positive bacteria, single-stranded DNA and double-stranded RNA found in some viruses, and beta-glucans found in fungal cell walls [6]. The innate immune system contains a series of pattern recognition receptors (PRRs), proteins that are able to recognize PAMPs; once activated, these PRRs elicit a series of cell signaling events that activate the cell’s specific effector functions that will be helpful to rid the host of the invading pathogen—namely, via the inflammatory response [6].

**Figure 2:**
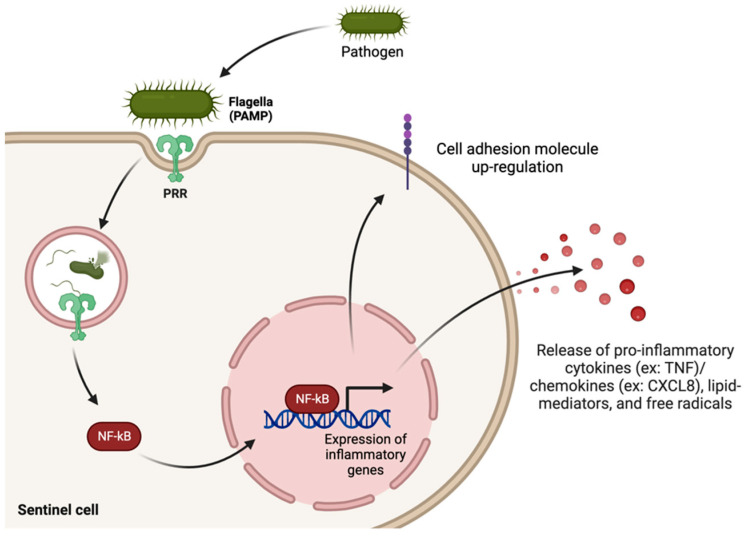
The innate immune system. Activation of the innate immune system begins with sentinel cell recognition of non-self. This usually occurs in the form of pathogen-associated molecular patterns (PAMPs)–pattern recognition receptor (PRR) interactions. Many downstream signaling cascades can become triggered by this PAMP–PRR interaction (which are cell- and pathogen-specific), but one of the most important signaling pathways that becomes activated is the nuclear factor kappa light chain enhancer of activated B cells (NF-κB) pathway. The effector molecules produced via this pathway are pro-inflammatory cytokines/chemokines, lipid mediators, and free radicals. Created in BioRender.com.

The inflammatory response is a crucial part of the body’s innate immune system, and when it acts acutely, it is very effective at clearing an infection. The cardinal signs of inflammation include redness, heat, swelling, and pain [9], and these are clinical manifestations of the protection offered by the innate immune system. The acute inflammatory response is dominated by neutrophils, white blood cells whose primary goals are to phagocytose the invading pathogen and to release pro-inflammatory mediators [10]. They are able to migrate to the site where the pathogen breached the first level of defense following the release of local mediators, such as histamine and nitric oxide, which function to increase blood vessel vasodilation [11]. Macrophages are another type of white blood cell that are also recruited to the site of injury to assist with pathogen removal. Pro-inflammatory mediators are produced via activation of several signaling pathways, the most prevalent of which is the nuclear factor kappa light chain enhancer of activated B cells (NF-κB) pathway [12]. The pro-inflammatory mediators produced include cytokines, chemokines, free radicals, and inflammatory lipid mediators (e.g., prostaglandins, leukotrienes) (See Figure 2). This pro-inflammatory response functions to clear pathogens; however, if this response persists for too long, then the tissues themselves can become damaged.

Maintenance of the innate immune system relies on immunological homeostasis, whereby the host’s pro- and anti-inflammatory responses balance one another in order to help return the host to baseline [13]. Therefore, resolution circuits are activated in order to ensure that this response and other aspects of the innate immune response act acutely and do not persist, and these circuits function to reduce neutrophil activation and increase macrophage efferocytosis of apoptotic neutrophils [14,15]. This is a critical step in the acute inflammatory response—if this inflammatory response either persists for too long or fails to clear the pathogen, chronic inflammation can and will ensue [10]. Because the innate immune system is always active within the host, it is typically not inhibited, but rather, it is regulated to help return the host to homeostasis; however, there are some ways in which the host can ensure its cessation as a backup mechanism [16]. For example, in addition to increasing IL-10 levels and the activity of anti-inflammatory T-cell subsets such as T-regulatory cells (Tregs), which both function to dampen the pro-inflammatory response, the innate immune system also seemingly modulates PRR expression on innate immune cells that favor inhibition rather than activation [16]. These inhibitory PRRs (iPRRs) are said to fine-tune the degree of innate immune cell activation via inhibition of cell signaling pathways [16].

If this innate inflammatory process is not successful at eliminating the body of the infection, then the adaptive immune response will become activated. The adaptive immune response primarily comprises B and T lymphocytes and memory functions. The activation of these lymphocytes leads to the production of antibodies (B cells) and increased macrophage signaling to clear pathogens (T cells).

## 4. The Adaptive Immune System and Response to Infection

The adaptive immune system is the last line of defense against invading pathogens. This system is triggered directly by T cell recognition of foreign invaders or by presentation of foreign antigen to T cells by one of the three antigen-presenting cells (APCs; monocytes/macrophages, dendritic cells, and B cells) [11]. APCs contain major histocompatibility complex (MHC) class II (MHC-II) on their surfaces, and these molecules are required for MHC-restricted antigen presentation to T cells and the subsequent activation of the adaptive immune response [17,18]. From here, the activated T cells can help stimulate other immune cells (including other types of T cells), can activate B cells to produce class-switched antigen-specific antibodies, and can clear the invading pathogen [11]. Finally, completion of this response allows for the development of immunological memory.

**Figure 3:**
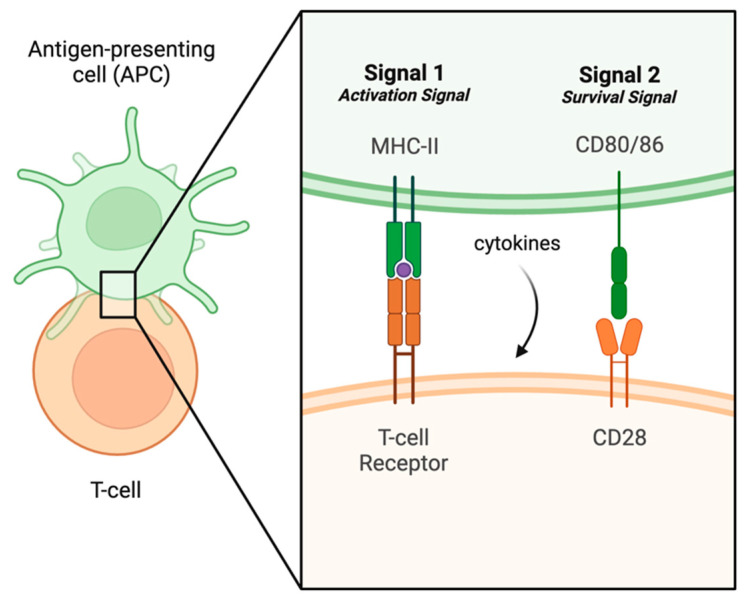
Peripheral tolerance and T cell activation. Activation of the adaptive immune system can begin with T-cell activation by an antigen-presenting cell (APC). This figure highlights the important concept of peripheral tolerance, or the two-signal hypothesis. Created in BioRender.com.

Overactivation of the adaptive immune system is commonly implicated in the development of autoimmune disease [19]. This is likely because the adaptive immune response is robust and produces memory. With that being said, there are many regulatory molecules in place to ensure proper functioning and activation (i.e., that T and B cells will not be inappropriately activated). One such mechanism occurs during T- and B-cell development: positive and negative selection (i.e., central tolerance) [20]. These selection processes occur during development to ensure that T and B cells both recognize foreign antigens but do not recognize foreign antigens too strongly [20]. Once these lymphocytes “pass” these selection processes, they are able to circulate in the body; however, there are more selection mechanisms in place in the periphery that the host subjects the naïve lymphocytes to (i.e., peripheral tolerance) [20]. For example, in order to ensure that activation of a naïve T lymphocyte is warranted, this activation process in the periphery requires two signals (See Figure 3).

The first signal is the “activation signal”, which comprises the interaction of the T-cell receptor (TCR) on the T cell with an MHC-II molecule on an APC [21]. The second signal is the “survival signal”, which comprises the interaction of the CD28 molecule on the T cell with a co-stimulatory molecule (CD80/86) on the APC [21]. Once the T cell becomes activated, it is able to carry out its effector functions, depending on what is needed at that time [21]. Typically, the T cell is “told” what is needed at that time for pathogen clearance via cytokines released by the APC onto the T cell [21]. These effector functions include the additional release of cytokines, activation of B cells, direct destruction of the pathogen, or activation of other cells or T-cell subtypes [21]. Additionally, some white blood cells have molecules expressed on their surfaces known as “immune regulatory molecules” or “checkpoints”, which can modulate T-cell functions [22]. These regulatory molecules are cytotoxic T-lymphocyte-associated protein 4 (CTLA-4), programmed cell death protein 1 (PD-1), and programmed death ligand 1 (PD-L1) [22]. CTLA-4 and PD-1 are both located on the surfaces of T cells and act as halting mechanisms of T-cell activation and function [23]. CTLA-4 interacts strongly with APC co-stimulatory molecules (i.e., signal two molecules), and this interaction inhibits T-cell activation and functioning [23]. Similarly, PD-1 can interact with PD-L1 on the APC’s surface, leading to T-cell anergy and exhaustion [23].

The adaptive immune response is maintained throughout the course of the host’s lifetime via the development of immunological memory. Following an adaptive immune response, memory T cells and memory B cells are created; upon repeated exposure to the same antigen in the future, these memory cells will be able to quickly and efficiently clear the pathogen [8].

## 5. Inflammation Resolution

Many studies have disproved the idea that resolution of inflammation (i.e., inflammation resolution) is a passive process, whereby the mere cessation of the inflammatory response is adequate enough to return the body to homeostasis [24]. It is now widely accepted that the resolution of inflammation is an active process, whereby the body synthesizes endogenous pro-resolving compounds and activates or suppresses certain cells/mediators, ultimately resolving inflammation without being immunosuppressive [25]. These pro-resolving compounds are known as specialized pro-resolving mediators (SPMs) [25], and they will be described in more detail toward the end of this article.

The goal of inflammation resolution is to allow the body’s tissues that have been harmed by a stimulus (e.g., pathogen, damaged tissue) or by the acute inflammatory response to the stimulus to return to homeostasis. Inflammation resolution is an active, coordinated process whereby certain molecular/cellular mechanisms take place in various immune cells in order to heal the harmed tissues [24]. One key event in this process is the recruitment and activation of macrophages to the site of infection [24]. As mentioned above, another key event in this process is the release of endogenous SPMs. Expectedly, the loss of or the dysregulation of inflammation resolution mechanisms results in pathological chronic inflammation and disease [24].

## 6. Mechanisms of Immunosuppression in Sepsis

**Figure 4:**
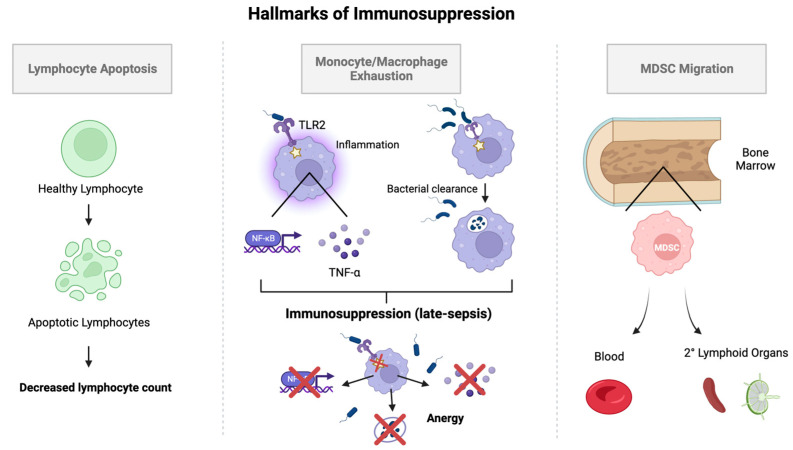
Mechanisms of sepsis-induced immunosuppression. Illustration of three major mechanisms underlying the pathophysiology of sepsis-induced immunosuppression: lymphocyte apoptosis, monocyte/macrophage exhaustion, and migration of myeloid-derived suppressor cells. Key: TLR2: Toll-like receptor 2; MDSC: myeloid-derived suppressor cell. Created in BioRender.com.

Three hallmarks of sepsis-induced immunosuppression include: monocyte/macrophage exhaustion, lymphocyte apoptosis, and myeloid-derived suppressor cell migration (See Figure 4).

### 6.1. Monocyte/Macrophage Exhaustion

**Figure 5:**
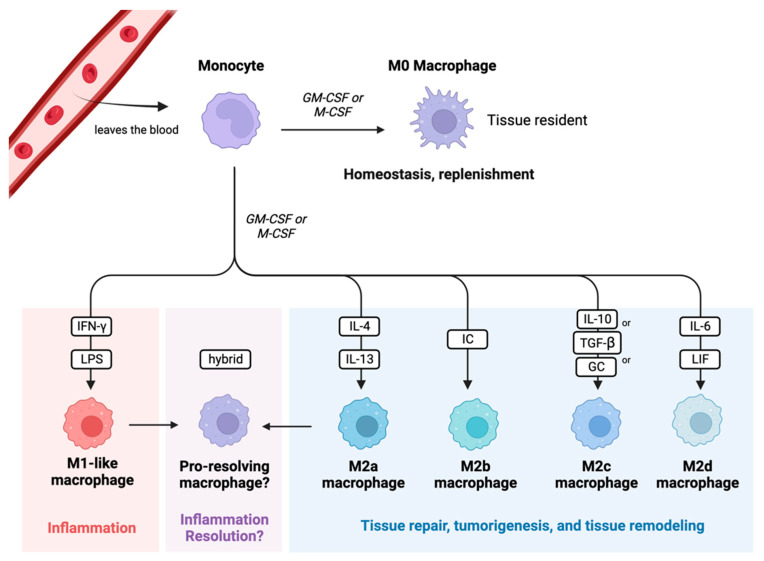
Monocyte polarization. This figure highlights the classical monocyte polarization scheme and classes of macrophages. Each class has a unique role within the immune system. Key: GM-CSF: granulocyte macrophage colony-stimulating factor; M-CSF: macrophage colony-stimulating factor; LPS: lipopolysaccharide; IC: immune complex; GC: glucocorticoid; LIF: leukemia inhibitory factor. Created in BioRender.com.

One hallmark of sepsis-induced immunosuppression is monocyte/macrophage exhaustion [3]. Monocytes are circulating white blood cells derived from the bone marrow and are major players in the innate immune system. Their primary role is to detect changes in homeostasis (usually caused by an infection and/or tissue damage) and respond to these changes by replenishing the pool of macrophages that are present in the tissues [26]. Once monocytes become activated, they turn into effector cells and produce cytokines, present antigens to adaptive immune cells, phagocytose, efferocytose, initiate inflammation, or resolve inflammation [26]. When monocytes initiate tissue inflammation, they exit the blood and enter the tissues at the site of the inflammation, thereby becoming macrophages. Macrophages can either be recruited to a specific tissue (which is what happens during the inflammatory response), or they can exist as residents within the body’s tissues. Macrophages can polarize into different subtypes depending on which subtype can best assist the host at that time. The previously accepted polarization classes are M0, M1-like, and M2-like; an M0 macrophage is a nonactivated tissue resident macrophage, an M1-like macrophage is a pro-inflammatory macrophage, and an M2-like macrophage is an anti-inflammatory macrophage (See Figure 5) [26]. It is classically understood that M1-like macrophages have increased production of pro-inflammatory cytokines, enhanced antigen presentation, and enhanced phagocytosis capabilities [27]. M2-like macrophages have diminished pro-inflammatory cytokine output and weakened antigen presentation, but enhanced phagocytosis capabilities [27,28]. These phenotypes can be artificially induced in vitro via monocyte stimulation, with LPS and IFN-γ leading to the development of a “classic” M1-like macrophage and IL-4 leading to the development of a “classic” M2-like macrophage [29,30].

M1-like macrophages are stimulated by IFN-γ and LPS; they are key initiators of the inflammatory response and are critical for the removal of bacteria [31]. On the other hand, M2-like macrophages are highly phagocytic and are key drivers of tissue remodeling following damage, tumor progression via the production of angiogenic factors, and parasite removal [31]. M2-like macrophages are also a main driver in the development of allergies. Additionally, the M2-like macrophage subtype can be further subclassified into M2a, M2b, M2c, and M2d [32,33]. These subtypes vary in what cytokines they release, what surface markers they express, and what specific roles they play within M2-like macrophage responses. M2a macrophages are stimulated via IL-4 and IL-13; they enhance cell growth and tissue repair and enhance macrophage endocytosis [32,33]. M2b macrophages are stimulated via immune complexes (IC); they are able to regulate the host’s immune status by secreting both pro- and anti-inflammatory cytokines [32,33]. M2b macrophages are also well known for their ability to promote tumor progression [33]. M2c macrophages are stimulated by IL-10, TGF-β, or glucocorticoids (GCs); like M2b macrophages, they are highly immune-regulatory but specialize in efferocytosis and phagocytosis of other apoptotic cells [32,33]. M2d macrophages are stimulated via IL-6 and leukemia inhibitory factor (LIF); they promote angiogenesis and tumor progression via the release of vascular endothelial growth factors and dampening of the immune response [32,33].

While the M1/M2 paradigm is helpful for investigators studying these different capabilities of macrophages in vitro, many researchers find that limiting the description of macrophage phenotypes to only M1-like or M2-like is not clinically relevant, as this tidy polarization process does not occur at a tissue level [29,30,34,35,36]. Current research explains that activated macrophages have high levels of plasticity and do not solely polarize into an M1-like or M2-like phenotype at the tissue level; rather, they adopt a phenotype that is highly dependent on both the environment they are in and their own ontogeny [29,30]. One such newly reported phenotype is the “pro-resolving” macrophage phenotype [29,30,37,38]. These dynamic, pro-resolving macrophages function primarily to resolve inflammation so that the host can return to homeostasis, while simultaneously contributing to wound/tissue repair [29,30]. Interestingly, these pro-resolving macrophages are said to have qualities of the classic M1- and M2-like phenotypes, with aspects that contribute to both pro-inflammatory and anti-inflammatory pathways [37]. Some studies even report that abnormal activation of this pro-resolving phenotype can lead to chronic organ dysfunction, highlighting their direct importance in the inflammation resolution process [39,40,41].

#### 6.1.1. PD-1 and PDL-1

Monocytes/macrophages contribute to sepsis-induced immunosuppression in a number of ways. Monocytes/macrophages can modulate the expression of their immune regulatory markers, an action that has implications for other immune cells and physiological pathways [42]. Changes in the expression of immune regulatory markers such as CTLA-4, PD-1, and PD-L1 can alter these interactions [22]. PD-1 (also referred to as CD279) is a transmembrane protein located on the surfaces of many immune cells; it is expressed in the highest concentration on activated T cells [43]. When PD-1 interacts with its ligand PD-L1, the T cell becomes anergic, or unresponsive to viable antigens [44,45,46,47]. PD-L1 is expressed by certain APCs (such as monocytes/macrophages) in inflammatory scenarios as well as by tumor cells [44]. Additionally, certain pro-inflammatory cytokines (especially IFN-γ) can trigger various signal transduction cascades within APCs, leading directly to increased expression of PD-L1 on their surfaces [48].

Physiologically, the PD-1/PD-L1 pathway aids in controlling the degree of inflammation at the tissue level by deactivating T cells that are directly contributing to the inflammation so as to protect tissues from immune-mediated damage [44]. Tumor cells overexpress PD-L1 in order to exist for longer periods of time without being “seen” by the adaptive immune system [44,48]. The PD-1/PD-L1 pathway has therefore become a major contributor to immunosuppression within the tumor microenvironment in many different types of cancers (notably melanoma and small-cell lung cancer) [49]. Understanding this major contribution of PD-1/PD-L1 to cancer-induced immunosuppression has led to the development of a new class of cancer immunotherapies called checkpoint inhibitors [22,49]. Due to many parallels existing between cancer-induced immunosuppression and sepsis-induced immunosuppression [3], checkpoint inhibitors were tested in clinical trials of sepsis patients and cecal ligation and puncture (CLP) sepsis models [50,51]. Hotchkiss et al. was the first to study the effects and safety profile of the anti-PD-1 immunotherapy agent nivolumab in a small clinical trial of sepsis patients [50]. More recently, Yang et al. showed that blocking PD-L1 on monocytes led to a dramatic increase in survival of CLP-septic mice [51].

Additionally, many researchers have assessed the potential correlation between immune regulatory marker expression and outcomes due to sepsis. Ruan et al. found that there is an increased number of cells expressing both PD-1 and PD-L1 in CLP-septic mice [52]. This finding was also true for humans, as there are reported increases in expression of both PD-1 and PD-L1 in human septic patients [52,53,54,55]. While changes in immune regulatory marker expression on monocytes/macrophages contributes to sepsis-induced immunosuppression, this finding is not specific to septic patients. As previously mentioned, cancer cells can implement similar changes in regulatory marker expression levels in order to evade the immune system. A more explicit example of monocytes/macrophages contributing to immunosuppression due to sepsis is monocyte/macrophage exhaustion [3].

#### 6.1.2. Exhausted Monocytes/Macrophages

Exhausted monocytes/macrophages are “hypoactive”, meaning that they have a decreased capacity to perform their normal effector functions when stimulated [56,57,58,59]. These normal effector functions include presenting antigens via HLA-DR, phagocytosing, efferocytosing, and releasing pro-inflammatory cytokines (e.g., TNF-α, IL-6, IL-8) via the NF-κB pathway [26]. Therefore, NF-κB status is an accepted marker of exhaustion [60]. As monocytes/macrophages from septic patients also have similarly reduced capacities of these normal functions, it can be inferred that septic monocytes/macrophages are exhausted [61]. A recent study revealed that septic macrophages had decreased pro-inflammatory cytokine production in response to various ligands, including bacteria and LPS [62]. In this study, the authors found that whole blood samples taken from 61 septic patients (blood taken within 24 h of meeting sepsis-3 criteria) had an overwhelming presence of inflammation, yet leukocytes from these samples had depressed responses to both LPS and bacteria [62], providing evidence that septic leukocytes were similar to the “exhausted” phenotype. This hypoactive phenotype present in septic monocytes/macrophages can be recapitulated in vitro by repetitively challenging monocytes/macrophages with isolated, low-dose bacterial endotoxins such as LPS [58,63].

It is important to briefly discuss here that monocyte/macrophage exhaustion is different from the phenomenon called endotoxin tolerance (ET). ET has many similar characteristics to exhaustion [61], and it is defined as reduced immune cell functional responsiveness to the bacterial endotoxin LPS after previously encountering LPS [56,57]. Blackwell et al. examined ET via monocyte cytokine production; they discovered that tolerized monocytes had impaired activation of NF-κB [64], which is also observed in exhaustion. Tolerized monocytes/macrophages exhibited a reduced capacity to react to subsequent LPS challenge; primarily of note was the drastic decrease in NF-κB-mediated TNF-α production upon LPS restimulation [65,66,67,68,69,70]. While the monocytes from late-septic patients seem to have many aspects similar to monocytes/macrophages experiencing ET, the conventional understanding of ET does not encompass the fundamental characteristics of immunosuppression observed in septic patients [59]. For example, monocytes/macrophages experiencing ET have enhanced bacterial phagocytosis, as it has been theorized that ET monocytes/macrophages adopt a phenotype similar to M2-like macrophages [56]. However, exhausted monocytes/macrophages and monocytes from septic patients demonstrate impaired bacterial phagocytosis, even though the mechanism underlying this decline is not known [71,72]. For these reasons, the emerging literature more accurately describes septic monocytes/macrophages as exhausted rather than tolerized [59]. However, the mechanism(s) underlying monocyte/macrophage exhaustion during the immunosuppressive stage of sepsis remains elusive.

Several possibilities for sepsis-induced monocyte/macrophage exhaustion include increased production of the anti-inflammatory cytokine IL-10, down-regulation of the NF-κB signaling pathway, decreased HLA-DR expression, and reduced lymphocyte signaling due to lymphocyte cell death [57,61,70]. Another possibility is the high mobility group box 1 (HMGB1) protein, which has been shown to increase macrophage pyroptosis [73,74,75], decrease macrophage efferocytosis ability [76], and enhance the suppressive activity of an immunosuppressive cell population called myeloid-derived suppressor cells (MDSCs) [77].

To overcome monocyte/macrophage anergy (i.e., exhaustion) several research teams have used IFN-γ in order to stimulate monocyte activity [78]. In one study performed by Döcke et al., the authors were able to increase HLA-DR expression in anergic monocytes, leading to sepsis resolution in eight of nine septic patients [78]. On the other hand, a more recent study showed that increased plasma IFN-γ levels were associated with secondary *Candida* sp. infections in late-sepsis patients [79]. Surprisingly, the authors also showed that IFN-γ reduced macrophage phagocytosis of zymosan particles [79]. Together, these two contradictory studies suggest that IFN-γ cannot be reliably regarded as an appropriate treatment to reverse sepsis-induced monocyte/macrophage exhaustion.

### 6.2. Lymphocyte Apoptosis

**Figure 6:**
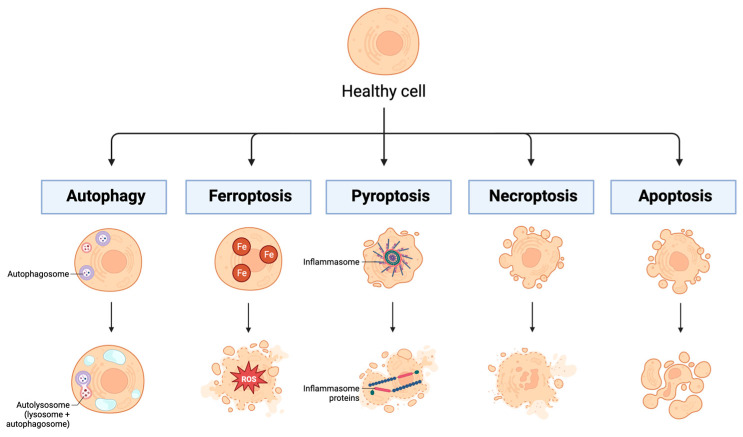
Mechanisms of regulated cell death. This figure highlights some important processes of regulated cell death: autophagy, ferroptosis, pyroptosis, necroptosis, and apoptosis. Key: Fe: iron; ROS: reactive oxygen species. Created in BioRender.com.

Another hallmark of sepsis-induced immunosuppression is lymphocyte apoptosis [3]. Before apoptosis can be discussed in more detail, it is important to mention that there are numerous types of regulated and unregulated cell death processes involving complex signaling cascades. The regulated and clinically relevant processes include, but are not limited to, autophagy, ferroptosis, pyroptosis, necroptosis, and apoptosis (See Figure 6). Autophagy is a process of regulated cell death by which autophagosomes collect cell waste products and deliver them to lysosomes for destruction [80]. This process is mainly employed to maintain homeostasis within the cell and to manage lipid metabolism [81]. Ferroptosis is a process of regulated cell death and is primarily caused by the accumulation of iron [82]. This accumulation leads to the development of reactive oxygen species (ROS), which contributes to both cell membrane rupture and lipid peroxidation [81]. Pyroptosis is a process of regulated cell death whereby destruction of the cell membrane is triggered by the activation of a multi-protein complex called the inflammasome [82,83]. Necroptosis is a process of regulated cell death that eventually leads to an influx of ions, swelling of the cell, and eventual breakdown of the membrane [82]. Due to many similarities between necroptosis and apoptosis (apoptosis is described in detail below), it has been suggested that necroptosis exists as a backup mechanism for apoptosis [81].

**Figure 7:**
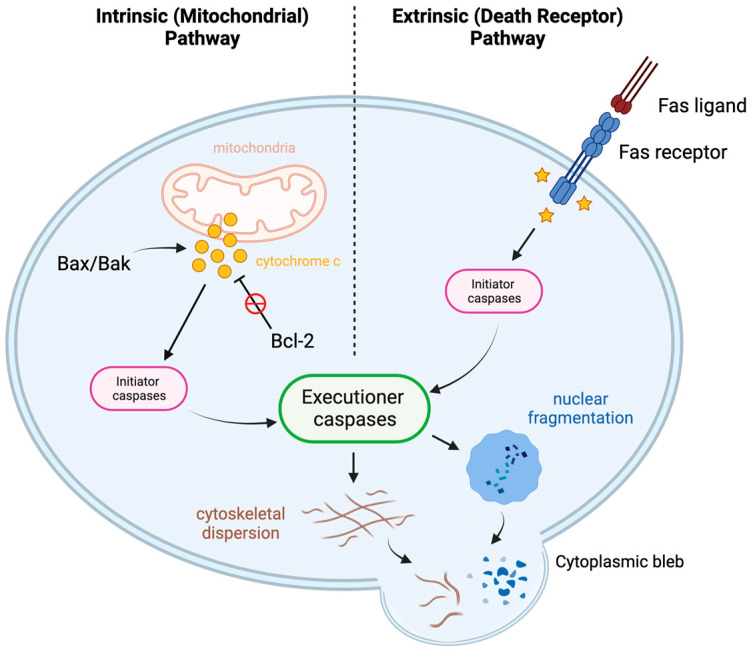
Apoptosis pathways. This figure demonstrates two pathways of apoptosis: the intrinsic (mitochondrial) pathway and the extrinsic (death receptor) pathway. Created in BioRender.com.

Apoptosis, or programmed cell death, is an active cellular process that is essential for proper functioning and regulation of many cells within the body, including immune cells. Apoptosis of faulty lymphocytes during and following lymphocyte development is crucial for proper immune functioning. There are two pathways that lead to lymphocyte apoptosis: “death by neglect” and “death by instruction” (See Figure 7) [84]. As discussed earlier, there are many mechanisms in place during lymphocyte development and activation to ensure proper performance and homeostasis within the population. If a lymphocyte fails to meet certain functional requirements, it will die via apoptosis (“death by neglect”). On the other hand, if a lymphocyte functions too aggressively, it will die via apoptosis (“death by instruction”). These apoptosis pathways that regulate lymphocyte development and expansion are necessary to ensure there are no self-reactive and/or hyporesponsive lymphocytes present in the immune system’s repertoire.

A defining feature of apoptosis is that the programmed cell death event does not trigger inflammation: the process occurs neatly and “quietly.” This highly coordinated pathway involves two main groups of players that work with each other in concert to ensure that this organized process remains balanced: apoptosis activators and apoptosis regulators. There are two main pathways of apoptosis activation: the extrinsic pathway and the intrinsic pathway [85]. Both pathways involve an apoptotic signal, which activates a proteinase called an initiator caspase, which in turn activates another proteinase called an executioner caspase [85]. The two pathways differ in how the initiator caspase becomes activated, but the end result remains the same. The extrinsic pathway, also known as the *death receptor pathway*, involves the Fas ligand (FasL) binding to a cell’s Fas receptor; the intrinsic pathway, also known as the *mitochondrial pathway*, involves the mitochondrial release of cytochrome c into the cytoplasm, which carries out effector functions necessary for apoptosis completion [86]. Two proteins that assist in the activation of apoptosis (and hence are considered “pro-apoptotic” proteins) are Bax and Bak, and their interaction with each other triggers destabilization of the mitochondrial membrane (via oligomerization) and the subsequent release of cytochrome c into the cytoplasm [85,87]. If Bax and Bak do not interact/oligomerize, the mitochondrial membrane remains stabilized, and there is no release of cytochrome c; if Bax and Bak do oligomerize, the membrane becomes destabilized, leading to the release of cytochrome c into the cytoplasm and completion of apoptosis [87]. Equally as important as apoptosis activation is the regulation of apoptosis by proteins within the Bcl-2 family. Bcl-2 is a regulatory protein within this family that functions as a direct regulator of Bax and Bak. Bcl-2 sequesters Bax and Bak so that they cannot oligomerize; this function earned Bcl-2 its “anti-apoptotic” title [86]. The level of Bcl-2, therefore, is a good biomarker to study changes in apoptosis within cells.

While some or all of the processes may be occurring in some way during the sepsis spectrum, they are difficult to definitively test for in a clinical setting. It is clear that cell death mechanisms are occurring in sepsis patients, as one of the main features of late sepsis is a decreased white blood cell count, and more specifically, lymphopenia [88]. For the purposes of this review, we will specifically focus on lymphocyte apoptosis, as that has been studied the most in experimental settings of late sepsis (i.e., in CLP sepsis models).

While lymphocyte apoptosis is a normal physiologic process necessary for proper immune functioning (i.e., a crucial part of the central tolerance of lymphocytes), this process can become dysregulated or can be hijacked by microbes, leading to an unnecessary loss of lymphocytes. Microbes can induce apoptosis in lymphocytes and other types of leukocytes in order to artificially create a more permissive environment for their survival within the host cells [89]. By triggering apoptosis within lymphocytes, microbes are able to thrive in the body because there are fewer immune cells capable of clearing them from the host. For example, *Bacillus anthracis*, the causative agent of anthrax, releases lethal toxin (LTx), which can lead to direct caspase release in macrophages, deceptively triggering their cell death pathways [90]. In the CLP model of polymicrobial sepsis, there is ~10% increase in the number of apoptotic lymphocytes compared with control mice [89]. Studies have shown that increased expression of Bcl-2 leads to increased survival of CLP-septic mice by ~40–50%, specifically through apoptosis prevention in splenic lymphocytes [89,91,92]. Additionally, there are numerous accounts confirming apoptosis of lymphocytes in CLP-septic mice using specific transferase-mediated dUTP nick-end labeling (TUNEL) staining methods [93,94,95].

Another mechanism of lymphocyte suppression that falls outside of, but is closely related to, the apoptotic pathways is the PD-1/PD-L1 pathway that was described earlier. While this pathway does not lead to apoptosis of lymphocytes, it renders the lymphocytes inactive, producing a very similar outcome. While the application of checkpoint inhibitors (i.e., PD-1 inhibitors) for sepsis-induced immunosuppression is still in its nascent stages, the enhancement of lymphocyte functionality in late sepsis may provide an exciting avenue for researchers moving forward.

In an attempt to target sepsis-induced immunosuppression, there have been several clinical trials whereby lymphocytes were targeted for restoration. In one study, recombinant GM-CSF or G-CSF was administered to late-septic patients in order to increase their lymphocyte counts [96]. These attempts initially seemed promising, but the beneficial effects did not persist long-term. In a similar clinical trial, IL-7 was administered to late-septic patients in an attempt to increase their populations of T lymphocytes, but the patients did not improve long-term [97]. In both of these instances, the failures were attributed to pathological migration and activation of myeloid-derived suppressor cells (MDSCs) [98]. It was postulated that MDSCs suppressed T lymphocyte activity and proliferation, negating the beneficial action of the intervention.

### 6.3. Myeloid-Derived Suppressor Cells

The third hallmark of sepsis-induced immunosuppression is increased myeloid-derived suppressor cell (MDSC) production/migration [3]. MDSCs are a group of heterogenous, immature cells of the myeloid lineage that work to suppress the innate and adaptive immune systems [99]. MDSCs can be divided into two main populations based on their lineage (although there are others that have been reported [100]): monocytic MDSCs (M-MDSCs) and polymorphic MDSCs (PMN-MDSCs) [99,101,102]. Both M-MDSCs and PMN-MDSCs are able to exert their inhibitory effects on the innate and adaptive immune systems in similar ways, with the primary mechanisms being via the release of ROS, production of arginase-1, and release of the anti-inflammatory cytokine IL-10 [99]. MDSCs exist in the bone marrow and are triggered to migrate into the blood and secondary lymphoid tissues via chemokine CXCL2 and IL-8 gradients [99]. Overall, MDSCs function as immunosuppressive cells, but their exact role and the meaning behind their migration patterns in patients with sepsis remains to be seen.

There is some controversy regarding the significance of the MDSC migration that is observed in septic patients [103,104,105,106]. Some studies report that the increase in MDSC migration out of the bone marrow in sepsis patients is partly responsible for the adverse clinical outcomes due to late sepsis [98,106,107,108]. In fact, Tang et al. suggests that inhibiting MDSCs would be one of the most effective treatments for sepsis-induced immunosuppression overall [108]. In septic patients, having elevated numbers of MDSCs in the peripheral blood was associated in some patients with longer ICU hospital stays and a higher statistical likelihood of acquiring a secondary infection [98]. In LPS-induced immunosuppression, MDSCs migrate from the bone marrow to the blood/secondary lymphoid tissues and inhibit the proliferation of lymphocytes [107]. Additional studies have shown that not only are MDSCs directly inhibitory on lymphocytes, but also their production of arginase-1 further disrupts T-cell functions [109]. Similarly, MDSCs can indirectly cause immunosuppression by activating Tregs [98]. This anti-inflammatory T-cell subset functions to inhibit other innate and adaptive immune cells, primarily via the release of IL-10 [98].

Conversely, there are also reports that MDSC migration during late sepsis could actually contribute to long-term survival in sepsis patients [103,104,106,110,111]. This paradoxical function of MDSCs was studied extensively in CLP-sepsis models. In one study, MDSCs were adoptively transferred into CLP mice early on in the infection timeline, and these mice had decreased mortality compared with sham animals [103]. In another study, mouse MDSC expansion was inhibited by pretreatment with the chemotherapeutic gemcitabine, and survival to CLP was dramatically reduced in the gemcitabine treatment group compared with the sham animals [104]. Similarly, in another study, inhibition of CLP mouse MDSC populations via GR-1 neutralization led to decreased survival compared with the sham animals [111]. In another infection model system, in vivo depletion of MDSCs in mice infected with *Trypanosoma cruzi* (the causative agent of Chagas disease) led to increased mortality compared with the sham animals [110].

A study by Schrijver et al. provides a strong case for the expansion and migration of M-MDSCs in particular correlating with improved survival in sepsis patients [112]. In this study, blood was taken from patients in eight ICUs with pneumonia secondary to sepsis [112]. The authors found that patients with high levels of M-MDSCs overall had significantly reduced 90-day mortality rates and improved survival compared with patients with high levels of PMN-MDSCs [112]. The contention is that due to the heterogeneity of MDSC populations, perhaps overall, MDSCs play a dual role during sepsis, with each unique MDSC population evolving over time after sepsis onset [103,112,113].

Overall, in sepsis patients, there is an elevation in MDSC numbers in the blood and the secondary lymphoid tissues. While a simple explanation would be that the migrating MDSCs are acting as the trigger for sepsis-induced immunosuppression, perhaps what is actually occurring here is that the host is attempting to correct the early hyper-proinflammatory phase of sepsis with MDSC migration. Due to unidentified dysregulation pathways in sepsis patients, perhaps the recruitment of MDSCs is not robust enough in these particular patients; therefore, the hyper-proinflammatory response persists until the late stage of sepsis sets in, and their presence then accentuates the immunosuppression. MDSC expansion and migration in sepsis appears to be a highly dynamic process, and these cells, conceivably, are malleable to the infection at hand. Although the complete consequences of increased MDSC production in sepsis have not been fully elucidated, their presence in the blood as a biomarker in the immunosuppressive phase of sepsis cannot be disputed.

## 7. Biomarkers

One of the most difficult aspects of developing sepsis therapeutics is the heterogeneity of the disease. There have been decades of failed sepsis therapeutics in clinical trials that were based on promising preclinical research. The discrepancy lies in the understanding that there are many factors contributing to patient outcomes due to sepsis, and the challenge in moving toward successful interventions stems from the variability of the immune system’s actions. The way the immune system responds to infection can be influenced by the stage of the inflammatory timeline in which it is acting. Therefore, the therapeutics administered in sepsis rely heavily on specific biomarkers, which allow physicians to decipher the immune status of their patients. Then, the physicians can tailor treatments more accurately and, in a way, more individually. For example, while common practice is to administer antibiotics in order to prevent detrimental outcomes due to sepsis, studies have shown that the antibiotics must be administered within the first hour of the sepsis diagnosis to provide any perceived benefit [114,115]; otherwise, administration of broad-spectrum antibiotics could actually facilitate the development of multi-drug-resistant infections, potentially causing an infection epidemic within the hospital [114]. Part of understanding the lack of favorable outcomes in sepsis due to antibiotic administration is appreciating that while microbial infections trigger sepsis, the main driver of sepsis is the patient’s own immune system—not the infection itself. Because sepsis itself is a clinical diagnosis (i.e., there is no “one test” that can diagnose sepsis), many times it either goes undiagnosed or it is diagnosed outside of the window of perceived benefit of antibiotic administration: once this window passes, the risk of antibiotic administration (i.e., antibiotic resistance) outweighs potential benefits [114]. The need for new, effective sepsis therapeutics will therefore depend heavily on quick thinking by the physician to test for specific biomarkers that could shed light on the patient’s immune status, as well as what therapeutic would best be warranted for administration at that time. While there likely will not be a single treatment that will be equally effective for every single patient with sepsis, an ideal treatment would be one that supports the immune system’s intrinsic ability to combat the pathogen while also resolving the infectious inflammation before it further causes damage to the body [116].

While there is no one test that definitively tells the physician that the patient has sepsis, there are certain reliable indictors that can shed light on a patient’s immune status. These measurable indicators, or *biomarkers*, are reproducible, highly sensitive, and highly specific [117]. Some biomarkers that are indicative of the early, hyper-proinflammatory phase of sepsis are similar to typical markers of inflammation: increased acute-phase reactant protein levels and increased pro-inflammatory cytokine/chemokine levels [117]. According to the Third International Consensus Definition for Sepsis and Septic Shock, elevated lactate is included as a biomarker for sepsis diagnosis [118], as elevations in lactate are correlated with increased mortality in sepsis [119]. Of note, one innate immune system protein called pentraxin 3 (PTX3) was recently assessed for its potential as a biomarker for early sepsis [120,121,122,123]. PTX3 is an acute-phase reactant that is highly expressed by phagocytes (such as monocytes/macrophages) following pro-inflammatory responses by the immune system [124]. PTX3 expression was reported in many studies to be upregulated in septic patients compared with non-septic patients, and this increase correlated with clinical severity and unfavorable prognosis [120,121,122,123,124,125,126].

Observation shows that most of the accepted sepsis biomarkers are indicators of the early, hyper-proinflammatory stage of sepsis. This likely is because many of the sepsis therapeutics in development focus on blunting the infection-triggered hyper-proinflammatory phase, with medications such as antibiotics, IL-6 inhibitors, and TNF-α inhibitors prevailing. The immunosuppressive stage of sepsis was not previously thought of as a potential area for therapeutic development, likely due to incomplete understanding of the underlying mechanisms leading to immunosuppression in sepsis. With that being said, accurate diagnosis of the late, immunosuppressive stage via biomarkers is critical for the proper administration of therapeutics that could modulate one of the three main underlying sepsis-induced immunosuppressive mechanisms (See Table 2).

### 7.1. IL-10

Two potential biomarkers of sepsis-induced immunosuppression that were already discussed in this review include decreased lymphocyte numbers in the blood (due to apoptotic mechanisms) and increased numbers of MDSCs in the blood and secondary lymphoid organs. Related to the aspect of immune cell exhaustion discussed earlier, another valuable marker of late sepsis that has been widely accepted is elevated levels of IL-10 in the serum [127]. Not only does the patient’s IL-10 level correlate with his/her sepsis severity score, but it also has been reported to be a major risk factor for increased clinical severity [128]. Many immune cells (both innate and adaptive) produce IL-10, as well as epithelial cells and keratinocytes [129]. Physiologically, IL-10 plays an important anti-inflammatory role in immune system homeostasis by balancing the pro-inflammatory response. IL-10 exerts its anti-inflammatory effect by inhibiting the release of pro-inflammatory cytokines, decreasing antigen presentation, and decreasing phagocytosis [129]. While IL-10 is crucial for normal immune cell function, sustained release of IL-10 can lead to immunosuppression by artificially inhibiting the normal functioning of immune cells. This understanding contributes to elevated blood IL-10 levels acting as a possible marker of sepsis-induced immunosuppression.

### 7.2. GPR18

More recently, the transmembrane protein GPR18 has been identified as a potential biomarker of sepsis-induced immunosuppression. Elevations in GPR18, a G-protein-coupled receptor found on immune cells and most abundantly in the spleen, has only recently been considered a marker of late sepsis [130]. GPR18 is the receptor for a specialized pro-resolving mediator (SPM) called resolvin D2 (RvD2) [130] (SPMs are discussed in detail below). In sepsis patients, elevated GPR18 expression on monocytes is correlated with a worse sepsis severity score [131]. Interestingly, low GPR18 expression on neutrophils is correlated with increased severity and a worse prognosis due to sepsis [132]. Taken together, these data provide evidence that GPR18 expression in different cell types may provide a way of “fine tuning” biomarkers in sepsis.

### 7.3. HLA-DR

Human leukocyte antigens (HLAs) are a family of genes of the MHC family that code for various proteins involved in the human immune system [133]. HLA-DR is an MHC class II glycoprotein complex expressed on APCs (monocytes/macrophages, dendritic cells, and B cells) [133]. Because HLA-DR is an MHC-II protein, it is required for adaptive immune system activation; therefore, researchers correlate increased expression of HLA-DR on APCs with a well-functioning immune system [3]. Recent research has suggested that decreased HLA-DR expression can be a reliable biomarker for sepsis-induced immunosuppression for many reasons. For example, one study reported that septic patients express 70% less HLA-DR than non-septic patients, and this decrease is associated with an increase in the patient’s sequential organ failure assessment (SOFA) score [134]. Similarly, another study found that decreased HLA-DR expression in immune cells within the bone marrow of septic patients was correlated with worse outcomes due to sepsis [135]. Another study found that following stimulation with LPS, immune cells with reduced expression of HLA-DR produce fewer pro-inflammatory cytokines than their immune cell counterparts with higher expression of HLA-DR [70]; this phenomenon is suggestive of immune cell exhaustion. Taken together, the results of these studies suggest that decreased levels of HLA-DR can serve as a potential biomarker of one of the three major mechanisms underlying sepsis-induced immunosuppression (monocyte/macrophage exhaustion).

**Table 2:** Biomarkers of Sepsis-Induced Immunosuppression.

Biomarker	Role and Correlation with Outcomes Due to Late Sepsis	References
Decreased lymphocyte count	Late-septic patients are reported to have a decreased lymphocyte count compared with non-septic patients. The decrease is thought to be contributed to inappropriate activation of apoptosis pathways. This biomarker is correlated with a higher likelihood of developing secondary infections.	[3,51,89,91,92,96,97]
Increased MDSC production/ migration	Late-septic patients have higher levels of MDSCs in the blood and secondary lymphoid organs compared with non-septic patients. While the purpose of increased MDSC production remains controversial, these cells are thought to contribute to worse outcomes due to sepsis due to their immunosuppressive and anti-inflammatory nature.	[3,98,103,104,106,107,108,109,110,111,112,136,137,138]
Increased IL-10	Late-septic patients have elevated levels of IL-10 compared with non-septic patients. Elevated anti-inflammatory responses triggered by IL-10 suggests that the body will not be able to mount an inflammatory response if the patient does develop a secondary infection. This biomarker is correlated with worse severity and worse clinical outcomes due to sepsis.	[3,127,128]
GPR18 expression	Late-septic patients have changes in GPR18 expression on their immune cells compared with non-septic patients. Changes to GPR18 expression suggests that there is decreased specialized pro-resolving mediator (SPM) bioavailability and uncoupled inflammation resolution circuits. Increased GPR18 expression on monocytes and decreased GPR18 expression on neutrophils is correlated with an increase in mortality due to sepsis.	[130,131,132]
Decreased HLA-DR expression	Late-septic patients have 70% less HLA-DR expression compared with non-septic patients. Decreased HLA-DR expression suggests that the immune system will be unable to activate T-cell-mediated adaptive immune responses if the patient develops a secondary infection. This biomarker is correlated with worse sequential organ failure assessment (SOFA) scores due to sepsis.	[3,70,134,135]

## 8. Specialized Pro-Resolving Mediators (SPMs) and Immunosuppression in Sepsis

As mentioned earlier, specialized pro-resolving mediators (SPMs) are a group of endogenously produced lipid mediators derived from fatty acids that work to resolve inflammation without being immunosuppressive [15,139,140,141,142,143,144,145,146]. SPMs are enzymatically derived from arachidonic acid (AA), eicosapentaenoic acid (EPA), and docosahexaenoic acid (DHA) [140]. Recent research has shown that SPMs are crucial for actively resolving acute inflammation following infection via interactions with their cognate receptors (found on many cellular targets, including immune cells) [15,140]. SPMs have been shown to resolve inflammation in many ways. One way is by enhancing macrophage functions during inflammation; SPMs have been shown to increase macrophage phagocytic and efferocytic abilities and dampen the macrophage-mediated release of pro-inflammatory cytokines [15,147]. On the other hand, SPMs reduce neutrophil activation and migration [15,148] while increasing their phagocytic ability [127,131]. Overall, they have been shown to mediate inflammation resolution by acting on immune cells (mostly on neutrophils and macrophages) to limit tissue damage and promote healing [140]. Defects in any SPM production pathway can lead to chronic inflammation and can contribute to immunopathology states, further highlighting the importance of endogenous SPM production in maintaining immune system homeostasis [140]. Taken together, SPMs resolve acute inflammation and prevent chronic inflammation.

Resolvins are derived from EPA (“E-series resolvins”) and DHA (“D-series resolvins”) [148], and their potent pro-resolving activities have been studied in preclinical murine models in the context of early infection or sepsis [144,145,149]. For example, Jundi et al. mapped resolution circuits in sepsis patients for two D-series resolvins, resolvin D1 (RvD1) and resolvin D2 (RvD2) [131]. They found from these studies that there is decreased bioavailability of endogenously produced SPMs present in these patients, ultimately leading to uncoupled inflammation resolution pathways in various immune cells [131]. When these septic patients were treated with ex vivo RvD1 and RvD2, their inflammation resolution pathways normalized [131]. Similarly, Dalli et al. reported that there was a measurable increase in SPM levels in the blood of sepsis non-survivors [150]. Like the conclusions drawn in Jundi et al., the authors suggest this may likely be a reflection of the dissociation of circulating SPMs and their receptors, leading to the uncoupling of inflammation resolution pathways [150].

While there are more studies in the literature that focus on the roles of SPMs in early sepsis, there are very few studies that focus on the role of SPMs in late sepsis. An established and accepted model of late polymicrobial sepsis is the two-hit model of CLP (hit one) followed by *Pseudomonas aeruginosa* secondary infection (hit two) [137,151,152,153]. This two-hit model enables the observation of how SPMs (administered late in an infection) may impact the host prior to a secondary bacterial lung infection [152,153]. In studies using this murine preclinical model, the administration of RvD2 increased secondary lung bacterial clearance, inflammation, and murine survival after secondary infection [152,153]. While the exact mechanism of this protection is currently unknown, it can be postulated that the SPMs positively enhance the function of certain immune cells such as monocytes, macrophages, and neutrophils in order to help clear the infection without triggering excess inflammation. This possibility is further demonstrated by studies showing that SPM administration lowers the amount of antibiotics needed in order for the host to successfully clear the bacterial infection or bacteria-produced biofilm [142,154,155,156]. Additionally, D-series resolvins were shown to directly increase macrophage phagocytic functions as a primary mechanism of clearing bacteria in late-sepsis murine models [153] as well as in cystic fibrosis murine models [157,158]. RvD2 was also reported to increase the number of M-MDSCs in CLP mouse spleens compared with sham spleens, and this increase was associated with enhanced bacterial clearance and reduced mortality due to sepsis [152]. These studies provide evidence that SPMs may have therapeutic value in the treatment of sepsis during the immunosuppressive phase.

## 9. Summary and Conclusions

In summary, three main mechanisms underlying sepsis-induced immunosuppression include lymphocyte apoptosis, monocyte/macrophage exhaustion, and MDSC migration. These mechanisms have one commonality: persistence of the early, hyper-proinflammatory phase of sepsis, which eventually leads to a late, immunosuppressive state. While there have been promising advancements in antibiotic development and preclinical study designs and research techniques, there is still a great need for effective sepsis therapies. Early recognition of late-sepsis biomarkers, such as decreased lymphocyte count, increased MDSC production, increased IL-10 levels, changes in GPR18 expression, and decreased HLA-DR expression, will help with targeted diagnoses and treatments. The use of SPMs is an intriguing possibility to protect the patient from secondary infections and death due to sepsis-induced immunosuppression. Further studies into the therapeutic value of SPMs in the treatment of sepsis-induced immunosuppression is warranted.

## Figures and Tables

**Table 1:** Innate vs. Adaptive Immune Systems.

	Innate Immune System	Adaptive Immune System
Response Speed?	Quick	Slow upon first exposure
Nonspecific or Specific?	Nonspecific	Specific
Development of Traditional Immunologic Memory?	No	Yes

## Data Availability

No new data were created in this study. Data sharing is not applicable to this article.

## Abbreviations

SPMs	specialized pro-resolving mediators
MDSCs	myeloid-derived suppressor cells
CRS	cytokine release syndrome
ILCs	innate lymphoid cells
PAMPs	pathogen-associated molecular patterns
PRRs	pattern recognition receptors
iPRRs	inhibitory pattern recognition receptors
LPS	lipopolysaccharide
NF-κB	nuclear factor kappa light chain enhancer of activated B cells
APC	antigen-presenting cell
TCR	T-cell receptor
IFN-γ	interferon gamma
TNF-α	tumor necrosis factor alpha
TLR2	Toll-like receptor 2
IL-4	interleukin 4
IL-6	interleukin 6
IL-7	interleukin 7
IL-8	interleukin 8
IL-10	interleukin 10
IL-13	interleukin 13
GM-CSF	granulocyte macrophage colony-stimulating factor
M-CSF	macrophage colony-stimulating factor
ICs	immune complexes
GCs	glucocorticoids
LIF	leukemia inhibitory factor
CTLA-4	cytotoxic T-lymphocyte-associated protein 4
PD-1	programmed cell death protein 1
PD-L1	programmed death ligand 1
CLP	cecal ligation and puncture
HLA-DR	human leukocyte antigen DR isotype
ET	endotoxin tolerance
HMGB1	high mobility group box 1
ROS	reactive oxygen species
Bcl-2	B cell lymphoma 2
FasL	Fas ligand
Bax	Bcl-2-associated X protein
Bak	Bcl-2 antagonist killer 1
LTx	lethal toxin
TUNEL	transferase-mediated dUTP nick-end labeling
G-CSF	granulocyte colony-stimulating factor
CXCL2	chemokine ligand 2
ICU	intensive care unit
M-MDSCs	monocytic MDSCs
PMN-MDSCs	polymorphic MDSCs
GPR18	G-protein-coupled receptor 18
RvD1	resolvin D1
RvD2	resolvin D2
PTX3	pentraxin 3
HLA	human leukocyte antigen
MHC	major histocompatibility complex
APC	antigen-presenting cell
SOFA	sequential organ failure assessment
AA	arachidonic acid
EPA	eicosapentaenoic acid
DHA	docosahexaenoic acid
